# Assessment of the behaviour and survival of nematodes under low oxygen concentrations

**DOI:** 10.1371/journal.pone.0197122

**Published:** 2018-05-14

**Authors:** Hiromi Kitazume, Mehmet Dayi, Ryusei Tanaka, Taisei Kikuchi

**Affiliations:** 1 Division of Parasitology, Department of Infectious Diseases, Faculty of Medicine, University of Miyazaki, Miyazaki, Japan; 2 Faculty of Forestry, Duzce University, Konuralp Campus, Duzce, Turkey; University of North Carolina at Chapel Hill, UNITED STATES

## Abstract

Oxygen is required for the completion of almost all known metazoan lifecycles, but many metazoans harbour abilities to withstand varying degrees and periods of hypoxia. *Caenorhabditis elegans*, one of the most popular model organism is extensively used as a model for the study of hypoxia and anoxia biology and it has been found that this nematode is capable of tolerance to varying degrees of hypoxia. Considering the extremely high diversity of nematodes, the effects of low oxygen concentration and mechanisms of adaptation to oxygen depletion differ among species. In this study, we used a simple assay to examine anoxia tolerance in four nematode species, including three free-living and one plant parasitic nematode. We found that the plant parasitic nematode *Bursaphelenchus xylophilus* can survive more than 14 days under anoxic conditions. Comparisons of behaviour during anoxia induction and the repertoire of oxygen sensation genes among the tested species suggested the existence of different oxygen sensation systems between *B*. *xylophilus* and *C*. *elegans*, which quickly introduce suspended animation in response to oxygen depletion to survive long-term anoxia.

## Introduction

Almost all metazoa require molecular oxygen for the completion of their lifecycles [1] (some rare exceptions have been reported [2]), tolerating varying degrees and durations of hypoxia. For example, metazoans occurring in the soil, deep sea or highlands can tolerate lower oxygen concentrations than those living on the Earth’s surface [1].

The free-living nematode *Caenorhabditis elegans*, inhabiting a high-organic matter environment, is a popular model organism for a wide array of biological and medical studies. *C*. *elegans*, like other animals, requires oxygen to develop and survive, but can maintain a normal metabolic rate at low oxygen concentrations of 3.6 kPa and has a near-normal metabolic rate at oxygen concentrations as low as 2 kPa [3, 4]. Interestingly, certain degrees of hypoxia (0.5% or 0.51 kPa oxygen) were reported to extend the lifespan of *C*. *elegans* [5]. Under lower oxygen concentrations (anoxia), however, the majority of nematodes died within 96–144 h [3].

*C*. *elegans* is a member of the phylum Nematoda, which consists of various species of roundworms (nematodes). Nematodes are the most abundant, ubiquitous and diverse multicellular organisms on Earth, found in every conceivable habitat, from the bottom of the deepest sea to near the top of the highest mountains as well as within animals and plants as parasites [6]. The oxygen concentrations preferred by different nematodes, therefore, vary among species and developmental stages. The maximum environmental partial pressure of oxygen normally experienced by nematodes likely occurs in aerated water and environments such as soil, deep sea and host bodies, provide lower oxygen availability [7, 8].

Most previous studies on nematode responses to hypoxia have been conducted with *C*. *elegans* (e.g. [3–5, 9, 10–12]) and little is known about the effects of low oxygen concentrations on other nematode species. In this study, we examined the effects of low oxygen concentrations using a simple and inexpensive method to generate anaerobic conditions in four distinct nematode species, including the model nematode *C*. *elegans* and three other developing laboratory models; *Pristionchus pacificus* (a bacterivorous species having a close association with beetles) [13], *Panagrolaimus superbus* (bacterivorous, having an unusual ability to survive extreme desiccation) [14], and *Bursaphelenchus xylophilus* (a plant parasite which can be maintained using fungi in the laboratory) [15]. We showed behaviour and survival of nematodes under anoxia condition can be easily tested using simple equipment and found that *B*. *xylophilus* is more tolerant of anoxia than the other species.

## Materials and methods

### Biological materials

*C*. *elegans* (strain N2), *P*. *pacificus* (strain RS2333) [13] and *P*. *superbus* (strain DF5050) [14] were cultured at 25°C on nematode growth medium plates seeded with *Escherichia coli* (strain OP50) according to standard culture techniques [16]. *B*. *xylophilus* (strain Ka4C1) was cultured on autoclaved barley grain inoculated with *Botrytis cinerea* at 25°C [15]. Culture was stopped at the vigorously multiplying phase to harvest fresh worms. Worm collections were performed using the Baermann funnel technique (for *B*. *xylophilus*) or by washing the plates (for the other species) and then the collected nematodes were washed twice with autoclaved water and once with M9 buffer solution [16] before use. To obtain age-synchronised nematodes for *C*. *elegans*, *P*. *pacificus* and *P*. *superbus*, eggs collected by bleaching gravid worms were incubated in M9 buffer at 25°C for 12 h. *B*. *xylophilus* eggs were collected as described in Iwahori and Futai [17] and were incubated in M9 buffer at 25°C for 12 h. n.b. *B*. *xylophilus* and *P*. *pacificus* hatch as the second stage larvae.

### Monitoring oxygen concentrations

Changes in gaseous and aqueous phase oxygen concentrations in a plastic bag were monitored every 10s using non-invasive O_2_ meter (Fibox 3 trace; TAITEC corporation, Saitama, Japan).

### Survivorship under anaerobic conditions

For mixed-stage assays, 10,000 worms were transferred to a 35-mm Petri dish containing 4 mL of M9 buffer and 40 μL of Gibco™ Antibiotic-Antimycotic solution (Invitrogen Corporation, Carlsbad, CA, USA) (water depth = ~2 mm). For age-synchronised worm assays, approximately 100 non-fed larvae (L1: *C*. *elegans* and *P*. *superbus*, L2: *B*. *xylophilus* and *P*. *pacificus*) or ~50 adult worms were transferred to a well of a 96-well plate containing 50 μL of M9 buffer and 1 μL of Gibco™ Antibiotic-Antimycotic solution (water depth = ~2 mm). Anaerobic conditions (oxygen concentration < 0.1% or 0.01 mg/L) were generated with a closed plastic bag (14 cm x 26 cm) and an oxygen scavenger (AnaeroPouch-Kenki; Mitsubishi Gas Chemical Company, Inc., Tokyo, Japan), which was originally developed to culture anaerobic bacteria.

Worm incubation under anaerobic conditions was started with 5 separate plastic bags and each opened on day 1, 2, 3, 4 or 14 post anaerobic induction to return them to normoxia and incubated for 24 h in M9 buffer without food. A worm that did not undergo any further development and was not moving at 24 h after the return to normoxic conditions was considered dead [3]. Control groups of worms were maintained in a plastic bag without oxygen scavengers or sealing. Incubation under a high concentration of carbon dioxide (20% CO_2_) was performed using a CO_2_ incubator (MCO-19AIC, Sanyo, Osaka, Japan). All the experiments were performed at 25°C.

The survival ratio was calculated by observing at least 100 nematodes at three random dish locations for mixed-stage, or all nematodes in the well for age-synchronised nematodes. No contamination by bacterial or fungal multiplication was detected during any assays. To assess reproductive ability of worms after anaerobic treatment, approximately 25 worms were transferred to growth media and growth was evaluated after 10 days of incubation at 25°C.

### Observation of nematode behaviour under anaerobic conditions

Nematode behaviour during the induction of anaerobic conditions was observed using an inverted light microscope and video monitoring system (Olympus IX71 microscope system; Olympus Corporation, Tokyo, Japan). Behaviour was observed at different time points (1, 2, 3, 4, 5, 6, 7, and 10 h) post anaerobic induction. Motility was evaluated by observing at least 100 individuals at three random dish locations each. During the observation the bags were kept closed and observations made from outside the bags.

### Identification of soluble guanylate cyclase genes and phylogenetic analyses

Orthologous *C*. *elegans* genes related to oxygen sensation (*gcy-31* to *-37*) were identified in other nematode species using the WormBase ParaSite database (http://parasite.wormbase.org/index.html) [18]. These genes were selected because each plays a role in the recognition of changes in oxygen concentrations. Amino acid sequences were aligned using MAFFT Multiple Sequence Alignment Software ver. v7.221 [19] and a maximum likelihood phylogenetic tree was produced using Randomized Axelerated Maximum Likelihood (RAxML) v8.2.7 phylogenetic analyses software [20] with 500 bootstrap replicates, based on the alignment applying the best-fitting substitution model identified with the option automatic protein model selection plus gamma (-m PROTGAMMAAUTO).

## Results

### Survivorship under anaerobic conditions

Changes of oxygen concentrations in a plastic bag with an oxygen scavenger were monitored by a non-invasive O_2_ meter. O_2_ concentrations in the bag started decreasing immediately after the insertion of an oxygen scavenger and reached less than 0.1% at 90 min of observation for both gaseous phase and the aqueous phase (S1 Fig). Once the bags were opened and the oxygen scavengers were removed, oxygen concentrations returned to normal (>21%) by 60 min and 80 min for gaseous phase and aqueous phase, respectively (S1 Fig).

**Fig 1:**
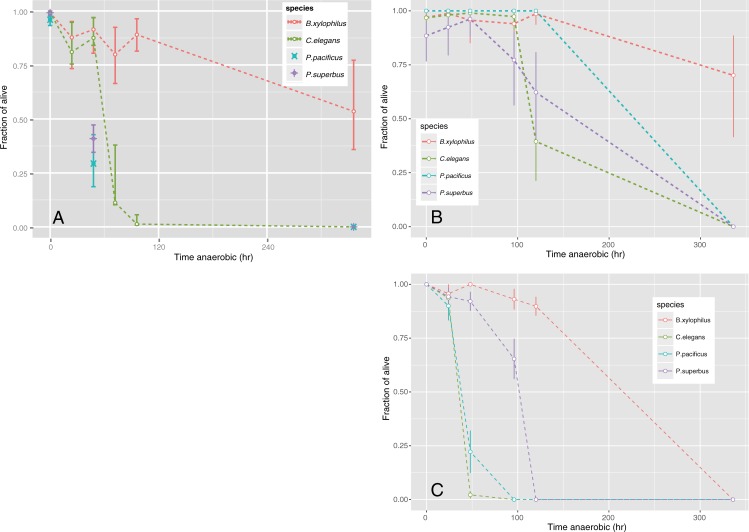
Survivorship of nematodes exposed to anaerobic condition. (A) survival of mixed-stage nematode exposed to anaerobic conditions for 24, 48, 72, 96 and 336 hours (B) non-fed larvae (*Caenorhabditis elegans* (L1), *Bursaphelenchus xylophilus* (L2), *Pristionchus pacificus* (L2) and *Panagrolaimus superbus* (L1)) were exposed to anaerobic conditions for 24, 48, 72, 96 and 336 hours. **(C)** adult nematodes: adult nematodes were exposed for 24, 48, 72, 96 and 336 hours. Error bars represent 95% CI estimated with a binomial model for each species using glmmML package implemented in R 3.2.4.

Survival of four nematode species under anaerobic condition were recorded for 1–14 days (24–336 hours) using mixed-stage nematodes (Fig 1A, S1 Table). After one or two days under anaerobic conditions, >80% of the *C*. *elegans* and *B*. *xylophilus* nematodes survived. However, significant differences were observed in those exposed to anaerobic conditions for >72 hours (two-sample *t*-test for equality of proportions, *p* < 0.05 for each time point). The survival ratio of *C*. *elegans* decreased to ~12% at 72 hours and <1% at 96 hours of exposure, respectively. In contrast, >80% of *B*. *xylophilus* nematodes survived 96 hours under anaerobic conditions. After 14 days (336 hours) under anaerobic conditions, the survival ratio of *B*. *xylophilus* was still >60% on average, whereas that of *C*. *elegans* was 0% (100% lethal). Survival of *P*. *pacificus* and *P*. *superbus* was tested after exposure to anaerobic conditions for 48 h and 14 days (Fig 1A, S1 Table). The survival ratios of both species after 48 h under anaerobic conditions were <50% and then decreased to 0% after 14 days, as observed with *C*. *elegans*. In the experiment, *B*. *xylophilus* worms that survived after 14 days anaerobic exposure were young larvae-biased. We therefore performed survival assays using age-synchronised worms.

The survival ratios of non-fed larvae (L1/L2) exposed to anaerobic conditions for 1–14 days (24–336 hours) are shown in Fig 1B (the original counts are in S2 Table). After 72 hours under anaerobic conditions, >90% of the *C*. *elegans* (non-fed L1) and *B*. *xylophilus* (non-fed L2) survived. As observed with the mixed-stage nematodes, there were differences of survival ratios in those exposed to anaerobic conditions for >72 hours (two-sample *t*-test for equality of proportions, *p* < 0.05 for each time point). The survival ratio of *C*. *elegans* decreased to ~40% at 96 hours post anaerobic induction. In contrast, >96% of *B*. *xylophilus* nematodes survived for 96 hours under anaerobic conditions. Survival ratios of *P*. *pacificus* (non-fed L2) and *P*. *superbus* (non-fed L1) after 96 hours under anaerobic condition were >70% and >60%, respectively. After 14-days (336 hours) exposure to anaerobic conditions, the survival ratio of *B*. *xylophilus* remained >70% on average, whereas no nematodes survived for *C*. *elegans*, *P*. *pacificus* and *P*. *superbus*.

The survival ratios of adult nematodes were lower than those of non-fed larvae (Fig 1C, S3 Table). Survival of nematodes at 24 hours post anaerobic induction was >90% for all the four species. But the survival ratio of *C*. *elegans* decreased to ~1% at 48 hours and no nematodes survived after 96 hours of exposure, which is consistent with the observations reported by previous studies [3, 21], whereas significantly high ratios of *B*. *xylophilus* (94.9%) survived at 96 hours post anaerobic induction (two-sample *t*-test for equality of proportions, *p* < 0.05). The survival ratios of *P*. *pacificus* and *P*. *superbus* after 48 hours under anaerobic conditions were >70%, and then decreased to 0% after 96 hours, as observed with *C*. *elegans*. No nematodes survived after 14 days (336 hours) for all species.

**Fig 2:**
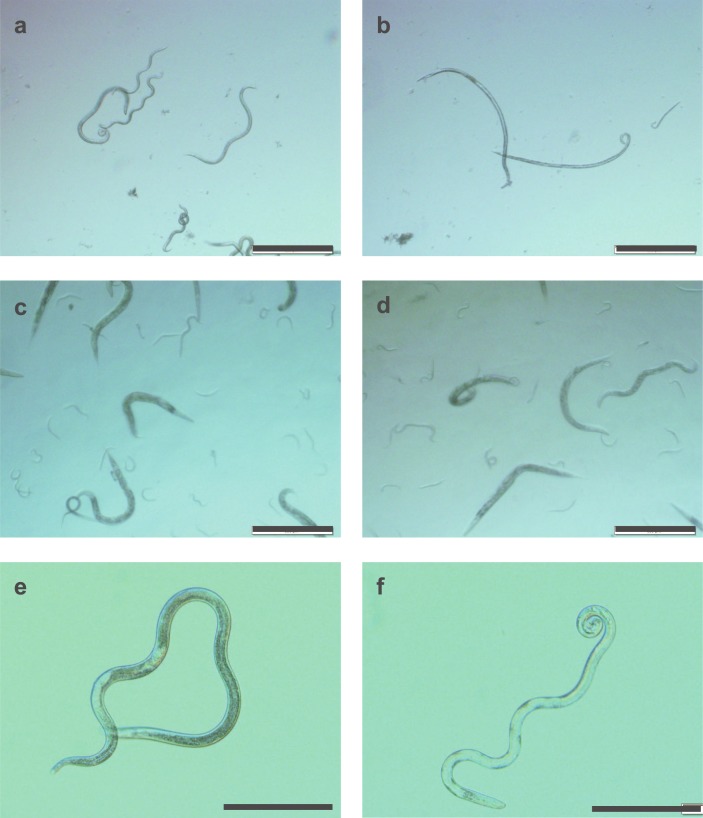
Appearance of nematodes under aerobic and anaerobic conditions. a: *Bursaphelenchus xylophilus* in M9 buffer before anaerobic exposure (0 h), b: *B*. *xylophilus* exposed to anaerobic conditions for 12 h, c: *Caenorhabditis elegans* before anaerobic exposure (0 h), d: *C*. *elegans* exposed to anaerobic conditions for 12 h. e: *B*. *xylophilus* recovered from 14 days of anaerobic exposure, f: *B*. *xylophilus* after 14 days of incubation in aerobic solution. Scale bars are 500 μm for a–d and 200 μm for e and f.

Of note, the survival ratio of *B*. *xylophilus* mixed-stages under anaerobic conditions for 14 days followed by 24 h aerobic return was significantly higher than the *B*. *xylophilus* controls, which were incubated under aerobic conditions for 15 days without food (40.23%) (S2 Fig) (two-sample *t*-test for equality of proportions, *p* < 0.05). In addition, after 14 days under anaerobic conditions, *B*. *xylophilus* retained high levels of lipid granules inside the body (Fig 2E) and locomotion was as vigorous as with freshly prepared nematodes, whereas the lipid granules were lost and movement was slow among the controls after 14 days (Fig 2F). To assess reproductive ability, *B*. *xylophilus* and *C*. *elegans* were each transferred to growth media after 14 days under anaerobic conditions. Nematode multiplication (>100×) and feeding behaviour were observed for *B*. *xylophilus*, but not for *C*. *elegans*.

The oxygen scavenger used in this study generates CO_2_ increasing its concentration in a plastic bag up to 20%. The effect of carbon dioxide on nematodes was examined in a 20% CO_2_ incubator (S3 Fig). The highest survival rates for both *C*. *elegans* and *B*. *xylophilus* were observed in the 20% CO_2_ group, followed by anoxia with CO_2_ and normoxia groups, suggesting no harmful effects of CO_2_ to the nematode survival. The higher survival ratios in 20% CO_2_ than in normoxia are probably because of the lower oxygen concentration the CO_2_ incubation derived, which leads to hypoxia life-span extension in nematodes [5].

### Nematode behaviour under anaerobic conditions

**Fig 3:**
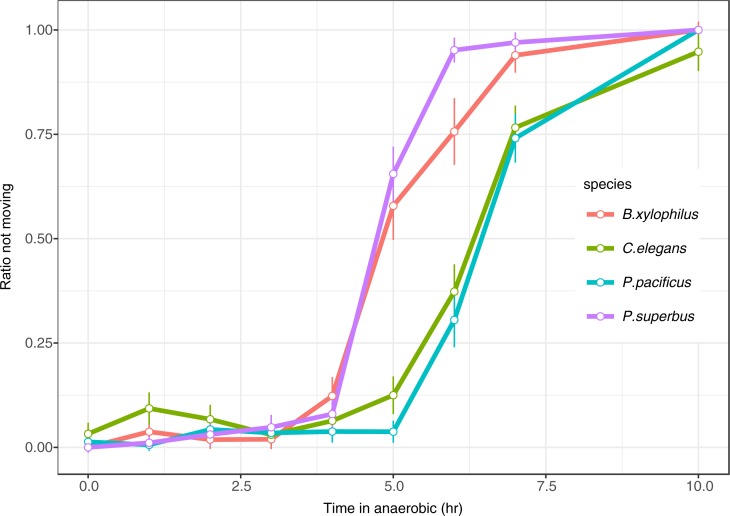
Ratio of nematodes in suspended animation phase during the induction of anaerobic conditions. Movement of mixed-stage *Caenorhabditis elegans*, *Bursaphelenchus xylophilus*, *Pristionchus pacificus* and *Panagrolaimus superbus* in M9 buffer under anaerobic conditions was recorded for 10 h. Error bars represent 95% confident intervals estimated as described in [22].

Nematode behaviour was monitored during the induction of anaerobic conditions (Fig 3, S4 Table). *C*. *elegans* locomotion became slower at ~10 h post induction, virtually stopping in the anaerobic condition. Approximately 75% of *C*. *elegans* ceased spontaneous movement after ~7 h of anoxia. On the other hand, 50% of *B*. *xylophilus* ceased spontaneous movement after 5 h of anoxia. Locomotion of *B*. *xylophilus* became slower at 4.5 h post induction and completely stopped at 10 h post induction (Fig 2A and 2B). Fig 3 shows the earlier slowing for *B*. *xylophilus* compared to *C*. *elegans* (4 vs. 5 h post induction). *P*. *pacificus* showed a highly similar pattern to *C*. *elegans* whereas *P*. *superbus* showed a similar pattern to *B*. *xylophilus* (Fig 3, S4 Table).

### Comparison of oxygen sensation genes

**Fig 4:**
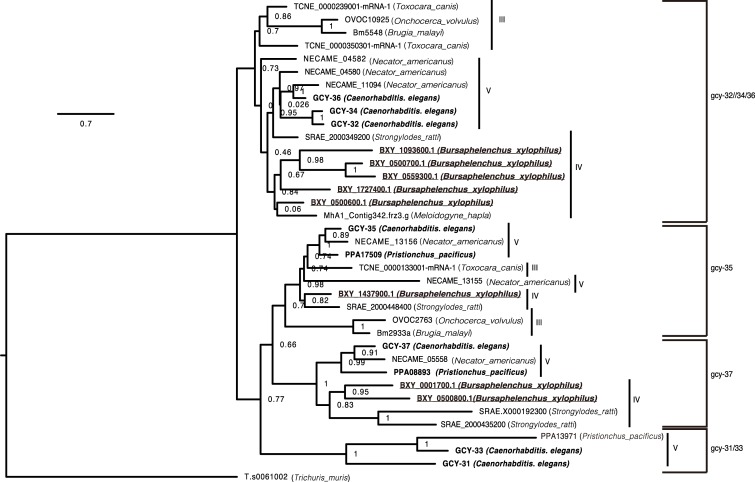
A maximum likelihood phylogenetic tree of the soluble guanylate cyclase (*gcy*) gene family (*gcy-31* to *gcy-37*) in nematodes. Values on nodes indicate the number of bootstrap trees of 100 bootstrap replicates, showing the split induced by the node. The scale bar shows the number of amino acid substitutions per site.

Next, the conservation of oxygen sensation genes in nematodes was investigated. In *C*. *elegans*, the soluble guanylate cyclase (*gcy*) gene family consists of seven genes (*gcy-31* to *gcy-37*) that are thought to play roles in oxygen sensation [9]. These genes were selected because they may play important roles in the recognition of changes in oxygen concentrations. A search of the WormBase ParaSite database [18] for homologues in the genomes of other nematode species identified 37 *gcy* orthologues from 10 nematode species, which included eight genes from *B*. *xylophilus* and three from *P*. *pacificus* (*P*. *superbus* genome information was not available). A phylogenetic tree of those genes from selected species contained four clusters when using a gene of the ancestral nematode (*Trichuris muris*) as an outgroup (Fig 4). The largest cluster contained *C*. *elegans gcy-32*, *gcy-34* and *gcy-36* (*gcy-32*/*34*/*36* cluster), and of two mid-size clusters, one contained *gcy-35* and the other *gcy-37* (*gcy-35* and *gcy-37* clusters, respectively). The cluster with *gcy-31* and *gcy-33* (*gcy-31*/*33* cluster) contained only three genes. In each cluster, with the exception of *gcy-31*/*33*, the species phylogeny was basically retained (i.e. genes from the same clade of nematodes were subclustered together in each cluster), although each cluster displayed some gene duplications or losses. Exceptions were observed in the *Necator americanus* genes NECAME04582 and NECAME13155 (Fig 4), which are likely truncated as they are much shorter (248 and 285 amino acids), than other sequences (e.g. lengths of *C*. *elegans gcy* genes range from 675 to 708 amino acids) to infer correct relationships. Gene duplications in *B*. *xylophilus* was observed in the *gcy-32*/*34*/*36* cluster, whereas no *B*. *xylophilus* genes were found in the *gcy-31*/*33* cluster.

## Discussion

In this study, the behaviour and survival capacity of four nematode species was examined under anaerobic conditions using a simple method that was originally developed to culture anaerobic bacteria. Although this method is relatively simple and requires no expensive equipment, it is considered reliable, as consistent results of *C*. *elegans* anoxia tolerance were obtained in the present and previous studies [3, 23]. The observation that non-fed or young larvae survived longer in anoxia than fed adult nematodes is also consistent with previous reports [3, 23]. Using this method, *B*. *xylophilus* was found to be highly tolerant to anaerobic conditions compared with the other tested nematode species. More than 50% of *B*. *xylophilus* mixed-stage larvae or non-fed L2 survived 2 weeks under anaerobic conditions, whereas none of the other nematode species survive the that period. This characteristic may reflect the *B*. *xylophilus* lifecycle, in which the nematodes experience very low oxygen conditions in host pine trees or vector insects [24], whereas the habitats of the other three nematode species are mainly soil, rotten fruits and dead insects [13, 14, 25].

Nematodes enter periods of arrested development to various degrees in response to environmental stimuli. For example, *C*. *elegans* enters a specialised formation called “dauer” in harsh environments [26]; the fungivorous nematode *Aphelenchus avenae* and the free-living nematode *P*. *superbus* achieve tolerance to desiccation by entering diapause phases [14, 27, 28]. Some parasitic nematodes, including *Ascaris* and *Meloidogyne* species, can survive for years when their hosts enter a diapause phase [29–31]. These developmental arrests are known to be regulated by a variety of environmental cues such as nutrient availability, overcrowding, desiccation, cool temperatures and host chemicals [26, 30, 32]. *C*. *elegans* has been reported to enter either suspended animation [4] or hypoxia-induced diapause [11] in response to decreased oxygen concentration.

*B*. *xylophilus* may more actively use oxygen deprivation as a cue to enter a quiescence phase for a long-term survival. Intriguingly, the survival ratios of the nematodes under anaerobic conditions were higher than those of the control group while maintained under aerobic conditions without food (n.b. normal life span of *B*. *xylophilus* with food at 25°C is about 25 days [33, 34], which is similar to *C*. *elegans* and *P*. *pacificus* [35–37]). As a possible explanation for this finding, under anaerobic conditions, *B*. *xylophilus* enters a quiescent phase with low energy demand, resulting in a longer life span. If the *C*. *elegans* death under anaerobic condition is because of energy deficiency and metabolic disorder due to continuous life activity regardless of the lack of oxygen, the *B*. *xylophilus* quiescence might be different from the ‘suspended animation’ reported in *C*. *elegans* as *B*. *xylophilus* survives notably longer in an anoxia condition than *C*. *elegans*. *B*. *xylophilus* ceases locomotion earlier than *C*. *elegans* when exposed to anaerobic conditions, indicating that *B*. *xylophilus* may have a specific or highly sensitive mechanism that senses anaerobic conditions and enters a quiescent phase to ensure long-term survival. However, inconsistently we observed *P*. *superbus* died much earlier than *B*. *xylophilus* in anaerobic condition although the nematode stopped locomotion as early as *B*. *xylophilus* did. Therefore, further studies are required to understand the mechanisms of this anaerobic tolerance (e.g. physiological or metabolic analysis using ATP/ADP ratio in low oxygen concentration etc.).

In *C*. *elegans*, GCYs participate in oxygen sensation by synthesising 3′,5′-cyclic guanosine monophosphate (cGMP) from guanosine-5′-triphosphate [9]. cGMP is a common second messenger in sensory transduction and has been implicated in oxygen sensation. GYCs are involved in the avoidance of hyperoxia and oxygen-induced aggregation and bordering, probably via the mediation of oxygen-sensing by URX, AQR and PQR sensory neurons [9]. By whole genome screening, the repertoire of *gcy* genes identified in *B*. *xylophilus* differed from that of *C*. *elegans*. Although there is a clear one-to-one orthologue relationship among genes in the *gcy-35* family, duplications of genes in the *gcy-32*/*34*/*36* and *gcy-37* families and those missing from the *gcy-31*/*33* family were observed in *B*. *xylophilus*. Structural, transcriptional and repertoire differences, as well as differences in the activation of subsequent pathways might be responsible for the distinct oxygen sensation ability of *B*. *xylophilus*. Therefore, it would also be interesting to further elucidate the molecular mechanisms underlying the ability of *B*. *xylophilus* to withstand anaerobic conditions.

The great majority of free-living eukaryotic organisms cannot withstand anoxic conditions for >24 h [38]. However, some animals are known to survive for long periods under anaerobic conditions [39]. For example, turtles (genera *Trachemys* and *Chrysemys*) survive up to 5 months and brine shrimp embryos (*Artemia franciscana)* up to 4 years under anaerobic conditions [38, 40, 41] through a glycolytic strategy (increasing the rate of glycolysis) and a metabolic depression strategy (depressing the rate of ATP use) [41]. As another example, members of the phylum Tardigrada have the ability to tolerate almost complete dehydration, and once dehydrated, tardigrades probably become tolerant to anaerobic conditions to withstand a wide range of physical extremes that normally disallow the survival of most organisms, such as extreme temperatures, high pressure and exposure to high doses of irradiation [42].

In this study, we found tolerance levels to anaerobic conditions and behaviour to oxygen varied among the nematodes, as those from environments different from that of *C*. *elegans* were more tolerant to extreme anoxia. Therefore, it would be interesting to examine behaviour and survivorship under anaerobic conditions using more species of nematodes, including many types of animal and plant parasitic nematodes, as well as those isolated from extreme environments, such as the deep sea. Although there are some previous reports on high anoxia tolerance of specific nematodes (e.g. [23, 43]), the equipment and methods used in those studies varied; thus, it is difficult to make direct comparisons. The simple method used in this study provides a good option to re-evaluate nematode tolerance and also screen new species under anoxic conditions.

## Supporting information

S1 Fig Change of oxygen concentration in a closed plastic bag by an oxygen scavenger. Gaseous phase and aqueous phase (M9 buffer) placed in the bag were monitored (A) from the insertion of an oxygen scavenger into the bag for 180 min, and (B) from bag opening and removal of the oxygen scavenger for 120 min after 24 h incubation with an oxygen scavenger. Each line represents an independent experiment.
(PDF)

S2 Fig Survivorship of control nematodes under aerobic conditions. Control nematodes were incubated in M9 buffer without food-supply at 25ºC in normoxic conditions. Error bars represent 95% confident intervals estimated with a binomial model for each species using glmmML package implemented in R 3.2.4.
(PDF)

S3 Fig Survival ratios of mixed-stage nematodes under different O_2_ and CO_2_ concentration. A) *B*. *xylophilus*, B) *C*. *elegans*.
(PDF)

S1 Table Number of mixed-stage nematodes survived after anaerobic exposure. (XLSX)

S2 Table Number of non-fed larvae survived after anaerobic exposure. (XLSX)

S3 Table Number of adult worms survived after anaerobic exposure. (XLSX)

S4 Table Number of move or stop nematodes exposed anaerobicc condition. (XLSX)
