# Supplementary material for: Assessment of the behaviour and survival of nematodes under low oxygen concentrations
Source: PLoS One. 2018 May 14;13(5):e0197122. doi: 10.1371/journal.pone.0197122 (PMC5951539; doi:10.1371/journal.pone.0197122)
Supplement: S1 Fig — Gaseous phase and aqueous phase (M9 buffer) placed in the bag were monitored (A) from the insertion of an oxygen scavenger into the bag for 180 min, and (B) from bag opening and removal of the oxygen scavenger for 120 min after 24 h incubation with an oxygen scavenger. Each line represents an independent experiment. (PDF) [file pone.0197122.s001.pdf]

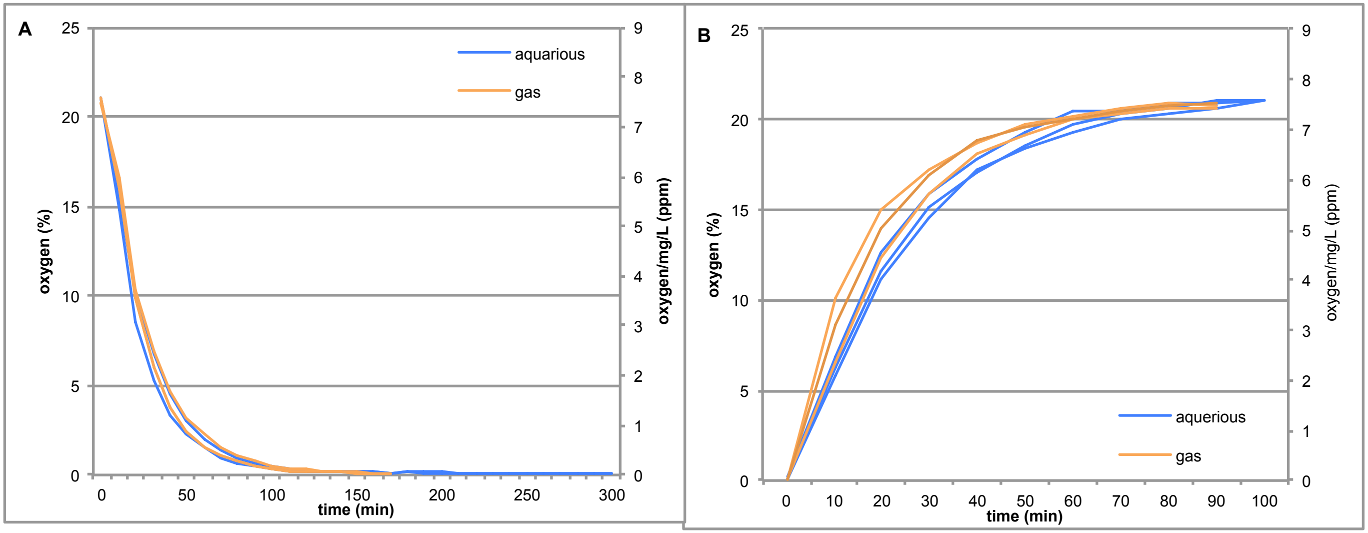


Fig S1. Change of oxygen concentration in a closed plastic bag by an oxygen scavenger. Gaseous phase and aqueous phase (M9 buffer) placed in the bag were monitored (A) from the insertion of an oxygen scavenger into the bag for 180 min, and (B) from bag opening and removal of the oxygen scavenger for 120 min after 24 h incubation with an oxygen scavenger. Each line represents an independent experiment.

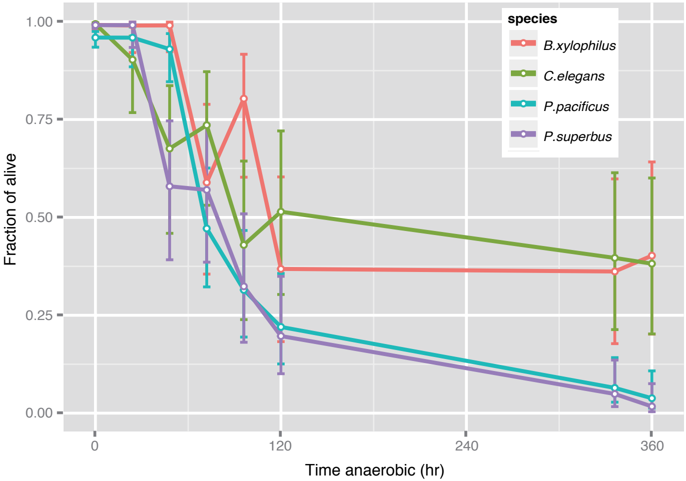


Fig S2. Survivorship of control nematodes under aerobic conditions. Control nematodes were incubated in M9 buffer without food-supply at 25ºC in normoxic conditions. Error bars represent 95% confident intervals estimated with a binomial model for each species using glmmML package implemented in R 3.2.4.

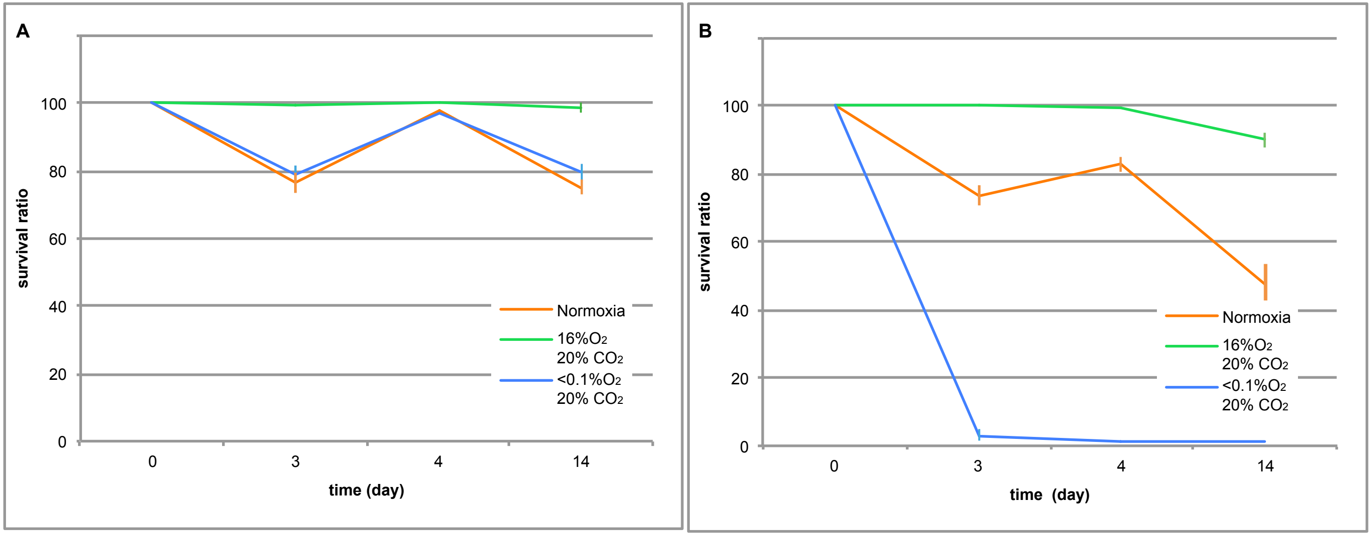


Fig S3. Survival ratios of mixed-stage nematodes under different O2 and CO2 concentration. A) B. xylophilus, B) C. elegans.
