# Supplementary figures and images for: Assessment of the behaviour and survival of nematodes under low oxygen concentrations
Source: PLoS One. 2018 May 14;13(5):e0197122. doi: 10.1371/journal.pone.0197122 (PMC5951539; doi:10.1371/journal.pone.0197122)

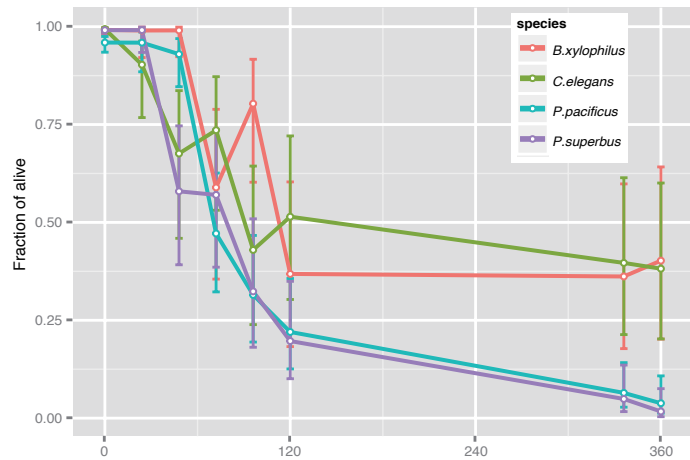

Supplement: S2 Fig — Control nematodes were incubated in M9 buffer without food-supply at 25ºC in normoxic conditions. Error bars represent 95% confident intervals estimated with a binomial model for each species using glmmML package implemented in R 3.2.4. (PDF) [file pone.0197122.s002.pdf]

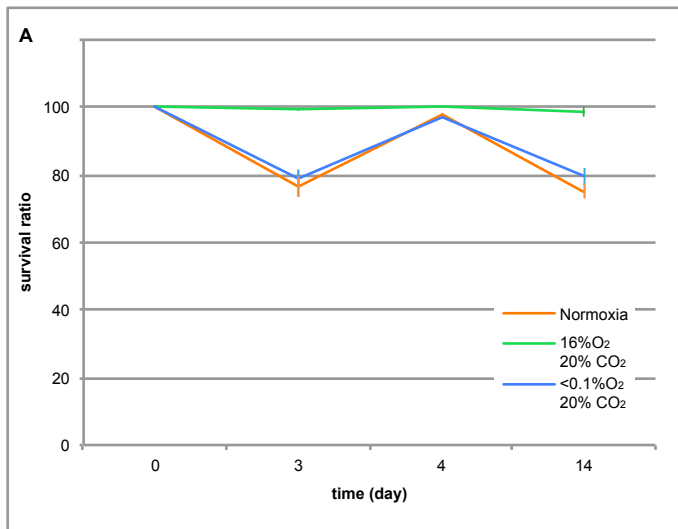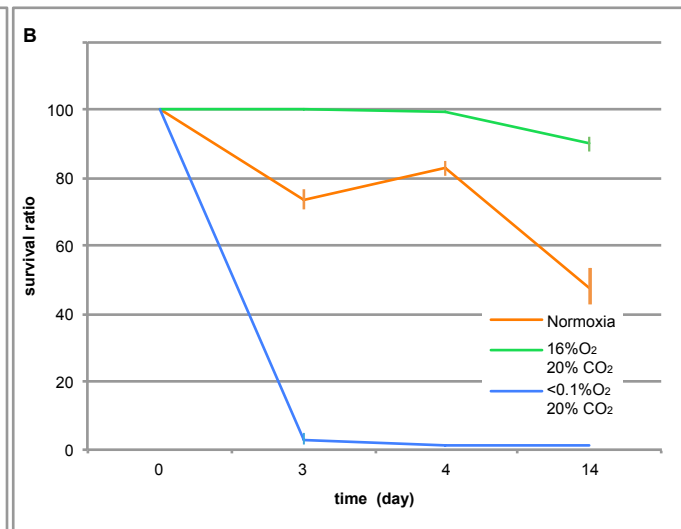

Supplement: S3 Fig — A) B. xylophilus, B) C. elegans. (PDF) [file pone.0197122.s003.pdf]
